# Characteristics and Glucose Uptake Promoting Effect of Chrysin-Loaded Phytosomes Prepared with Different Phospholipid Matrices

**DOI:** 10.3390/nu11102549

**Published:** 2019-10-22

**Authors:** Seong-Min Kim, Jae-In Jung, Changhoon Chai, Jee-Young Imm

**Affiliations:** 1 Department of Foods and Nutrition, Kookmin University, Seoul 02707, Korea; ksm618@nate.com (S.-M.K.); shinseo23@naver.com (J.-I.J.); 2 Department of Applied Animal Science, Kangwon National University, Chuncheon 24341, Korea; chchai@kangwon.ac.kr

**Keywords:** chrysin-loaded phytosome, soya phosphatidylcholine, egg phospholipid, aqueous solubility, glucose uptake, C2C12 muscle cells

## Abstract

Chrysin-loaded phytosomes (CP) were prepared using either soya phosphatidylcholine (SPC) or egg phospholipid (EPL) by the solvent evaporation method. Different phospholipid matrices resulted in significant differences in size, mechanical property and solubility of the CP. The most stable CP was obtained with EPL at a molar ratio of 1:3 (chrysin: EPL, CEP-1:3). CEP-1:3 displayed an average size of 117 nm with uniform size distribution (polydispersity index: 0.30) and zeta potential of −31 mV. A significantly greater elastic modulus of CEP-1:3 (2.7-fold) indicated tighter packing and strong molecular bonding than those of CP prepared with SPC (CSP-1:3). X-ray diffraction and Fourier transform infrared spectroscopic analysis of CEP-1:3 confirmed molecular complexation. CEP-1:3 displayed a greater glucose uptake promoting effect than free chrysin and CSP-1:3 in muscle cells by stimulating gene expression of peroxisome proliferator-activated receptor γ and glucose transporter type 4. The results of the present study suggest that the phospholipid matrix used for the preparation of phytosomes critically influences their performance.

## 1. Introduction

The prevalence of type II diabetes has been continuously increasing, and the development of safe and effective antidiabetic agents is becoming more important [1]. Flavonoids are the most abundant dietary compounds and they exert an antidiabetic effect by modulating a variety of cellular processes, such as insulin secretion in pancreatic β-cells, glucose homeostasis in the liver, and glucose uptake in skeletal muscle [2]. Since skeletal muscle plays a paramount role in the uptake and utilization of glucose, skeletal muscle and nuclear hormone receptors have been suggested as key targets for glycemic control [3].

Chrysin (5,7-dihydroxyflavone) is often found in nature, such as in flowers and propolis. Its antidiabetic and anti-inflammatory activities have been reported [4,5]. Chrysin effectively suppressed oxidative damage and helped to recover motor and sensory functions in a spinal cord injury rat model [6]. In addition, it has been suggested as an anti-amyloidogenic and neurotrophic agent, and successfully suppressed age-related macular degeneration in rats [7,8].

However, the oral bioavailability of chrysin is as low as 0.003%, and it is rapidly excreted from the body in the feces [9]. Thus, the development of an efficient delivery system is urgently required to improve the bioavailability and overall efficacy of chrysin. In previous studies, chrysin-loaded polymeric nanoparticles produced using poly (lactic-co-glycolic) acid exerted a greater chemotherapeutic effect on human gastric cells than that induced by free chrysin [10]. Chrysin nanoemulsion more strongly stimulated apoptosis in MCF-7 breast cancer cells via inhibiting the nuclear factor erythroid 2-related factor 2 pathway [11].

Phytosome is a type of nano-delivery system which consists of molecular-level complexation between phytochemicals and phospholipids [12]. Phytosomes are markedly smaller than liposomes and have been suggested as a promising strategy for improving the bioavailability of phytoconstituents [13]. Soya phosphatidylcholine (SPC) is a purified phospholipid widely used for the preparation of phytosomes. Also, egg phospholipid (EPL) contains various phospholipid species, such as sphingomyelin, phosphatidylethanolamine, and lysophosphatidylcholine, in addition to phosphatidylcholine. The profile, position, and saturation level of fatty acids esterified in the glycerol backbone also showed significant differences between SPC and EPL [14]. These differences in the phospholipid matrix may influence the structure, bioactivity and performance of phytosomes.

In the present study, chrysin-loaded phytosomes (CPs) were prepared using either SPC or EPL, and the physicochemical characteristics of CPs were examined. Also, the effects of CP on glucose uptake and the expression of related genes were analyzed using C2C12 skeletal muscle cells.

## 2. Materials and Methods

### 2.1. Materials

SPC (>98% purity), EPL (phosphatidylcholine 84.5%, phosphatidyl-ethanolamine 9%, sphingomyelin 3%, lysophosphatidylcholine 3%, lysophosphatidyl-ethanolamine 0.5%) were kindly provided by Doosan Corporation (Seoul, Korea). 2-(N-(7-nitrobenz-2-oxa-1,3-diazol-4-yl)amino)-2-deoxyglucose (2-NBDG) was obtained from Cayman Chemical (Ann Arbor, MI, USA). Chrysin and the solvents used in this study were purchased from Sigma-Aldrich Inc. (St. Louis, MO, USA). Taqman® Universal master mix, Taqman^®^ probes (5′-fluorescein based reporter dye; 3′-TAMRA quencher) and the high capacity RNA-to-cDNA kit were purchased from Applied Biosystems (Foster City, CA, USA).

### 2.2. Preparation of CP

The CP was prepared using the solvent evaporation method at a molar ratio (chrysin:phospholipid) of 1:2 and 1:3 using SPC or EPL, respectively. Briefly, chrysin (159 mg, 50 mM) and SPC or EPL (969 and 1453 mg, 100 and 150 mM) were dissolved in 12.5 mL tetrahydrofuran and stirred for 4 h at 40 °C. The solvent was removed using a rotary evaporator (Eyela, Japan) at 40 °C. CP in a round bottom flask was recovered using 12 mL distilled water. The empty phytosome was prepared through the same procedure without the addition of chrysin.

### 2.3. Characterization of CP

#### 2.3.1. Particle Size, PDI, and Zeta Potential

The mean particle size, PDI, and zeta potential of the CP were measured by dynamic light scattering using a zeta potential analyzer (ELS-1000ZS, Otsuka Electronics, Osaka, Japan) at 25 °C.

#### 2.3.2. Entrapment Efficiency of CP

Entrapment efficiency (EE) of CP was determined as described by Babazadeh et al. [15]. Briefly, CP (5 mg chrysin/mL) was mixed with an equal volume of ethanol and was subjected to filtration equipped with an Amicon Ultra-15 centrifugal filter (MW cutoff: 3 kDa, Millipore, Darmstadt, Germany). The samples were subjected to centrifugation at 7500 × g for 20 minutes at room temperature. The untrapped free chrysin was quantified from the filtered phase using HPLC (1260 infinity II, Agilent; Waldbronn, Germany). For the quantification of chrysin, samples (20 μL) were injected into a C18 column (4.6 × 250 mm; YMC, Japan) and eluted with a mobile phase consist of methanol:acetonitrile:water:acetic acid (40:30:30:0.1). The flow rate was 1 mL/minute, and detection was made at 320 nm using a UV detector. The amount of chrysin was calculated using a standard curve constructed with a chrysin standard. EE was calculated as follows:

EE (%) = (the amount of entrapped chrysin/the amount of chrysin addition) × 100%.

#### 2.3.3. Solubility

The solubility of samples was determined by the method of Telange et al. [16] with slight modification. Excess amounts of samples were dispersed in PBS (pH 6.8) at 37 °C under agitation and were subjected to centrifugation at 5000 × g for 15 minutes. The supernatant was obtained by filtering samples through a 0.45 μm PVDF membrane filter (CNW Technology, Korea). The content of chrysin in the supernatant was quantified using a plate reader at 320 nm and a standard curve constructed with chrysin.

#### 2.3.4. Topographical Analysis and Measurement of Elastic Modulus

Aliquots of CSP-1:3 (chrysin-loaded phytosome prepared with soya phospholipid at the molar ratio of 1:3) and CEP-1:3 (chrysin-loaded phytosome prepared with egg phospholipid at the molar ratio of 1:3) (approximately 3 μL) were placed on a microscope slide (75 × 26 mm; LK Lab, Korea) and dried at room temperature. The topography of samples were analyzed using atomic force microscopy (AFM; Easyscan 2, Nanosurf AG, Liestal, Switzerland). The dried sample was scanned in a non-contact mode with a PPP-NCLR silicon cantilever probe with a nominal spring constant of 48 N m^−1^ (Nanosensors, Nanoworld AG, Neuchatel, Switzerland). The area (50 × 50 μm) of the scanned sample and topographical data obtained from the AFM analysis were processed using the specialized image processing software SPIP (Image Metrology, Lyngby, Denmark). AFM (EasyScan 2) equipped with a colloidal probe (colloid size: 10 μm) with a gold coating on the reflex side and a spring constant of 0.14 N m^−1^ (Shocong; AppNano Inc., Mountain View, CA, USA) was used to measure the elastic modulus of samples [17]. The elastic modulus was calculated by fitting the force-versus-distance curves to the Derjaguin–Muller–Toporov model [18]. Measurement of the elastic modulus was performed in triplicate.

#### 2.3.5. FTIR

A FTIR-spectrophotometer (Vertex 70; Bruker, Billerica, MA, USA) equipped with an attenuated total reflectance (ATR) was used to investigate molecular interactions involved in complex formation between chrysin and EPL. Vacuum-dried samples were scanned over the infrared range (4000 to 600 cm^−1^) and characteristic peaks were compared.

#### 2.3.6. XRD

The XRD patterns of chrysin, EPL, the physical mixture of chrysin, EPL, and CEP-1:3 were measured using an X-ray diffractometer (SmartLab, Rigaku, Tokyo, Japan). The X-ray diffractograms were obtained at 45 kV tube voltage at room temperature with a scanning diffraction angle (2 θ) ranging from 5 °C to 80 °C and a step size of 0.2 °C.

#### 2.3.7. SEM

Samples dried under vacuum were placed on an electron microscope brass stub and coated with gold in an ion sputter. Representative images of samples were taken and diameters of particles were calculated using JEOL 7610 f-plus SEM (Tokyo, Japan) at 5.0 KV.

#### 2.3.8. In Vitro Release

The in vitro release of CP was analyzed using the dialysis method [19] with slight modification. Samples (equivalent to 2 mg chrysin) were solubilized in 2.5 mL methanol and placed in a dialysis bag (MW cut off; 12–14 KD, Spectrum Laboratories Inc., Rancho Dominguez, CA, USA). A releasing medium (100 mL PBS, pH 6.8) containing tween 80 (1%, *v*/*v*) was used for the dialysis. The release experiment was done in a 37 °C water bath under agitation. At given time intervals, the releasing medium (1 mL) was taken and replaced with an equal volume of fresh medium. The content of chrysin in the medium at the designated time intervals was assayed at 320 nm using a standard curve constructed with chrysin.

### 2.4. Bioassays

#### 2.4.1. Cell Culture

The C2C12 myoblasts were obtained from ATCC (Manassas, VA, USA) and cultured as described elsewhere (Kim et al., 2017). The cytotoxicity of samples was determined using MTT [3-(4, 5-dimethylthiazol-2-yl)-2, 5-diphenyltetrazolium bromide] assay.

#### 2.4.2. Glucose Uptake Assay Using C2C12 Skeletal Muscle Cells

Two sets of samples were prepared. In the first set, fully dissolved chrysin using DMSO (1%, *v*/*v*) and CEP-1:3 containing an equivalent amount of chrysin were introduced to C2C12 cells for 24 h. In the second set, samples were obtained after filtration of 1 mg/mL chrysin (without DMSO) and CEP-1:3 through a 0.45 μm syringe filter. The effect of samples on glucose uptake was determined in C2C12 skeletal muscle cells as described by Kim et al. [20]. Briefly, C2C12 cells grown in glucose free DMEM were incubated for 24 h in the presence of sample and 2-NBDG (100 μM). After washing 3 times with PBS, the fluorescence intensity of cellular 2-NBDG was quantified at excitation of 485 nm/emission of 535 nm using a microplate reader (Biotek Instruments Inc., Winooski, VT, USA).

#### 2.4.3. RNA Extraction and Quantitative Real Time PCR (qRT-PCR)

qRT-PCR was performed using a StepOne Plus™ real-time PCR system (Applied Biosystems) to analyze the effect of samples on the expression of genes associated with glucose uptake in C2C12 muscle cells. Cell harvest, total RNA extraction, and qRT-PCR were conducted as described elsewhere [21]. The primers used in the analysis were as follows: β-actin (Mm00607939_s1), PPARγ (Mm01184322_m1), GLUT4 (Mm00436615_m1). The gene expressions of PPARγ and GLUT4 was normalized to that of β-actin. The relative level of gene expression was determined using the comparative C_T_ (the value of the threshold cycle) method using the StepOne Plus software.

### 2.5. Statistical Analysis

The results were expressed as the mean ± standard deviation, and all quantitative determinations were carried out at least in triplicate. One-way analysis of variance (ANOVA) was conducted using the SPSS statistical software (SPSS Inc., Chicago, IL, USA). Duncan’s multiple comparison test was used to examine significant differences among groups when a significant difference (*p* < 0.05) was found through ANOVA.

## 3. Results and Discussion

### 3.1. Formation of Chrysin-Loaded Phytosomes (CPs)

CPs were prepared using either SPC or EPL at a molar ratio (chrysin:phospholipid) of 1:2 and 1:3, respectively. Stable CPs were not produced at the 1:1 ratio with neither of the matrices, and separation of yellowish crystalline chrysin precipitates was observed after removal of the solvent. Separate precipitation of chrysin was probably attributed to incomplete complexation with phospholipids. Thus, CPs produced at the ratio of 1:1 were excluded from further experiments. The entrapment efficiency of CP was approximately 99% and it was not influenced by the stoichiometric molar ratios under the tested conditions (Table 1). The high encapsulation efficiency suggests that high affinity for the formation of CPs. The affinity of flavonoids for phospholipid vesicles may depend on the structure of the flavonoids. The planar structure of chrysin formed by a double bond between C-2 and C-3 facilitates interaction between chrysin and phospholipids [22].

### 3.2. Size, Polydispersity Index (PDI), and Zeta Potential of CPs

The average particle size of the CPs was in the range of 89 to 134 nm and CEP appeared to have more uniform particle size distribution (small PDI) than that observed for CSP (Table 1). The average particle size of CPs was comparable to that reported in previous reports (range: 50–153 nm) [15,23,24]. The zeta potential, an index of colloidal stability, increased in parallel with the molar ratio of phospholipid increased in both CSP and CEP. The high negative zeta potential values were probably due to the formation of the hydroxyl group of hydrophilic shell and the presence of negatively charged phospholipid in the aqueous environment [25,26]. Particle size and zeta potential are regarded as major contributors to the stability and reproducibility of phytosomes. The zeta potential varies depending on the surface charge of dispersion, and higher values imply greater physical stability. At least ± 30 mV are required to prevent flocculation and maintain stable dispersion [19]. The average particle size and zeta potential of CEP-1:3 was not changed after 3 weeks of refrigerated storage, indicating the absence of significant coagulation in the main particle population. Nanoparticles greater than 100 nm are preferentially uptaken by the lymphatic system and highly negatively charged nanoparticles facilitate lymphatic transport [27].

Nara et al. [28] compared the oxidative stability of aqueous micelles and liposomes prepared from SPC and egg phosphatidylcholine. They reported that egg phosphatidylcholine had Sn1-16:0 and Sn-2-18:1 as the major fatty acid, while SPC had Sn1,2-18:2 fatty acids as the most abundant component. In this regard, the differences in the fatty acid profiles of phosphatidylcholine may influence the oxidative stability of phytosomes during long-term storage.

### 3.3. Solubility of CPs

**Figure 1:**
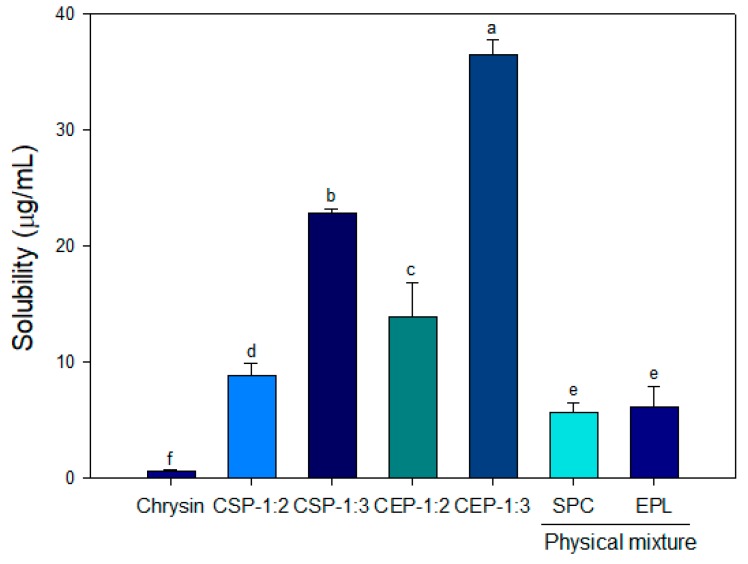
Aqueous solubility of chrysin-loaded phytosomes prepared from soya phosphatidylcholine and egg phospholipid. CSP-1:2 and CSP-1:3, chrysin-loaded phytosomes prepared with soya phosphatidylcholine at the molar ratio of 1:2 and 1: 3, respectively. CEP-1:2 and CEP-1:3, chrysin-loaded phytosomes prepared with egg phospholipid at the molar ratio of 1:2 and 1:3, respectively. Data are expressed as the mean ± standard deviation. Bars with different letters shows significant differences at (*p* < 0.05).

The aqueous solubility of CPs was determined using pH 6.8 phosphate-buffered saline (PBS) buffer to simulate the gastrointestinal environment. When chrysin was dispersed in PBS buffer, it was barely soluble in the medium and the calculated solubility after centrifugation was approximately 0.6%. The solubility of CPs was significantly improved in both types of phospholipid matrix, and they exhibited greater solubility compared with that of the physical mixture (*p* < 0.05). The solubility of CPs was significantly increased in parallel with the molar ratio of phospholipid, and the solubility of CEP-1:3 was improved by 60-fold compared with that of free chrysin (Figure 1).

The reduced crystallinity and impartment of the amphiphilic nature are responsible for the improved solubility [29,30]. Phospholipids have the ability to form spontaneous self-assembled structures in an aqueous medium. Moreover, the phospholipid complexation of chrysin may facilitate the entry of chrysin into intestinal brush borders. Curcumin-loaded phytosomes showed improved absorption, and their half-life was increased by 1.5-fold in rats [31]. In addition, supplementation of the curcumin phospholipid complex provided protection from extensive hydrolytic digestion [32]. Based on the previous particle characterization and solubility results, CEP-1:3 was selected as an optimum condition for the preparation of CP and further characterization was conducted.

### 3.4. Topographical Analysis of CPs Using AFM 

**Figure 2:**
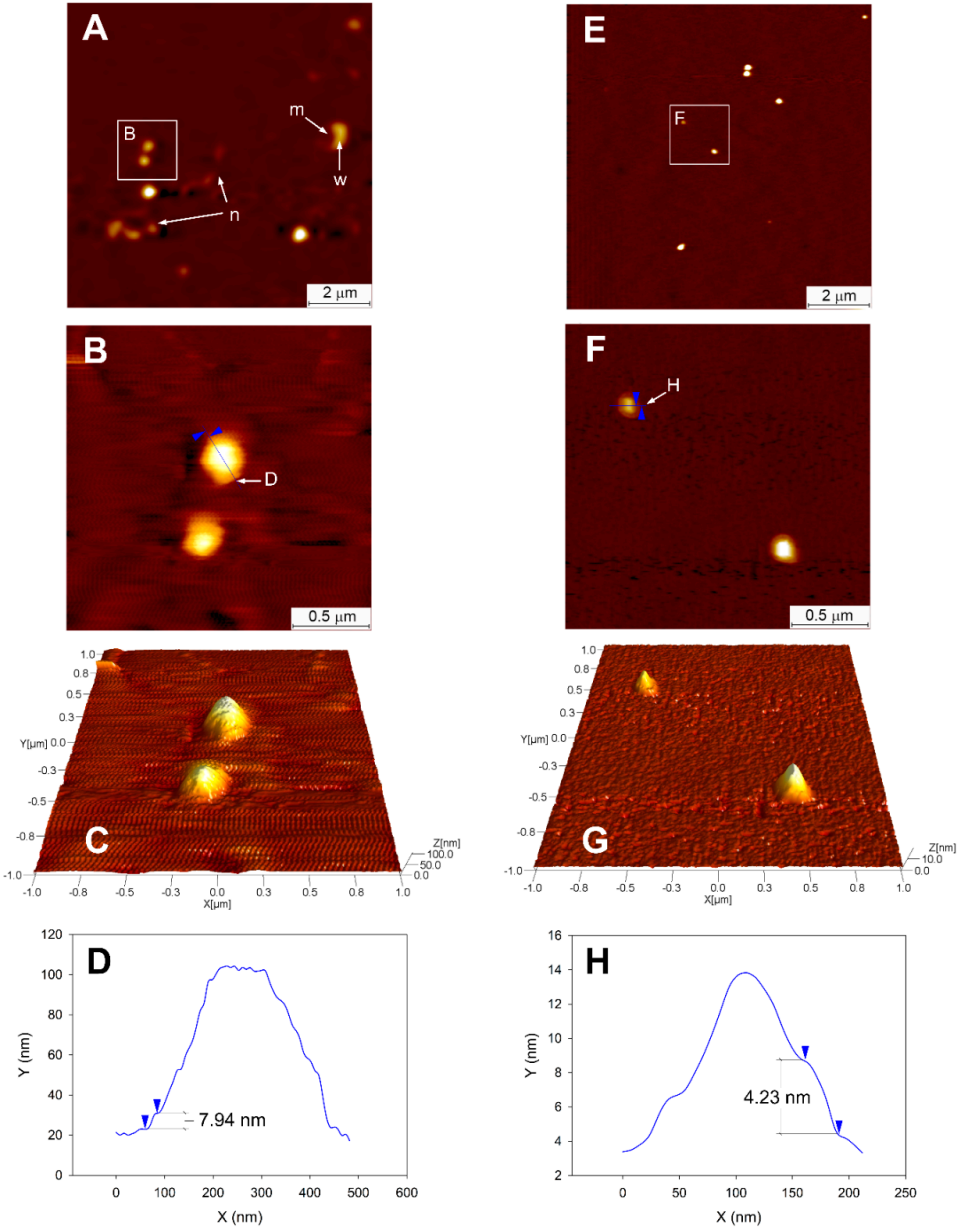
Atomic force microscopy image of (**A**) CEP-1:3 and (**B**) CSP-1:3. CSP-1:3 and CEP-1:3 adsorbed on a microscopic slide were analyzed using AFM in a non-contact mode. (**B**) and (**F**) are magnified AFM images of the area indicated by the solid white squares in (**A**) and (**E**), respectively. (**C**) and (**G**) are three dimensional images of **(B)** and **(F)**, respectively. (**D**) and (**H**) are cross-sections following the line in (**B**) and (**F**), respectively. Cursors indicated in (**B**) and (**F**) geometrically correspond to those in (**D**) and (**H**), respectively. “m” and “n” indicate a ruptured CSP-1:3 and debris of torn CSP-1:3. “w” indicates a part of ruptured CSP-1:3 (m) bearing remnant water.

AFM analysis of samples was performed in a non-contact mode that measured the height of particles via vibrational contact of the AFM probe with the particles. Two-dimensional and three-dimensional images of CSP and CEP prepared at a 1:3 ratio (CSP-1:3, CEP-1:3) were obtained after adsorption of samples on a glass surface (Figure 2). Ruptured (indicated as “m”) and debris (indicated as “n”) particles were often found in CSP-1:3. Some remnant water possibly released from “m” was visualized on the right side of “m”. In contrast to CSP-1:3, “m” and “n” were not observed in CEP-1:3. 

The rupturing and tearing of CSP-1:3 may be related to physical strength. The elastic modulus of CSP-1:3 and CEP-1:3 was approximately 12 and 33 kPa, respectively, and the elastic modulus of CEP-1:3 was 2.7-fold greater elastic modulus than that of CSP-1:3 (Table 2). The higher elasticity of CEP-1:3 provided greater resistance on the physical stress exerted by the AFM probe. This result suggests that the presence of different phospholipid species in EPL may facilitate tighter packing and strong molecular interactions in structure formation.

Magnified AFM images of CP indicated that the particles were sphere shaped with rims at the edges. The rims of CSP-1:3 and CEP-1:3 were pointed with blue cursors, and the height of the rims was estimated following cross-sections indicated in the AFM images (Figure 2B,F). The calculated height of the rim of CSP-1:3 and CEP-1:3 was 7.94 and 4. 23 nm, respectively (Figure 2D,H). The rims may be formed by the gravitational collapse of particles on microscope slides and thus, the edges of the particles may be folded and overlapped. The heights of rims should be twice of the thickness of the CSP and CEP membranes.

It was reported that the head-to-tail length of a phospholipid is approximately 2 nm [33]. Moreover, the heights of the rims of CSP-1:3 and CEP-1:3 are approximately four- and two- fold greater than the typical length of phospholipids, respectively (Figure 2D,H). Based on this speculation, the membranes of CSP-1:3 and CEP-1:3 are likely to exhibit a bilayer (liposome-like) and monolayer (micelle-like) structure, respectively. The bilayer structure of CSP-1:3 may allow more water to be included in the particles than the monolayer structure of CEP-1:3 and this may explain the easy rupturing and tearing of CSP-1:3.

The phytosome has been reported as a liposome like bilayer structure conjugated with phytoconstituents [13]. However, its structure and mechanical properties may vary according to the type of phospholipid matrix used for its preparation. This study is the first to report EPL consisting of different phospholipid species was able to form more stable and elastic vesicles than that produced with pure SPC.

### 3.5. Fourier Transformation Infrared Spectroscopy (FTIR) 

**Figure 3:**
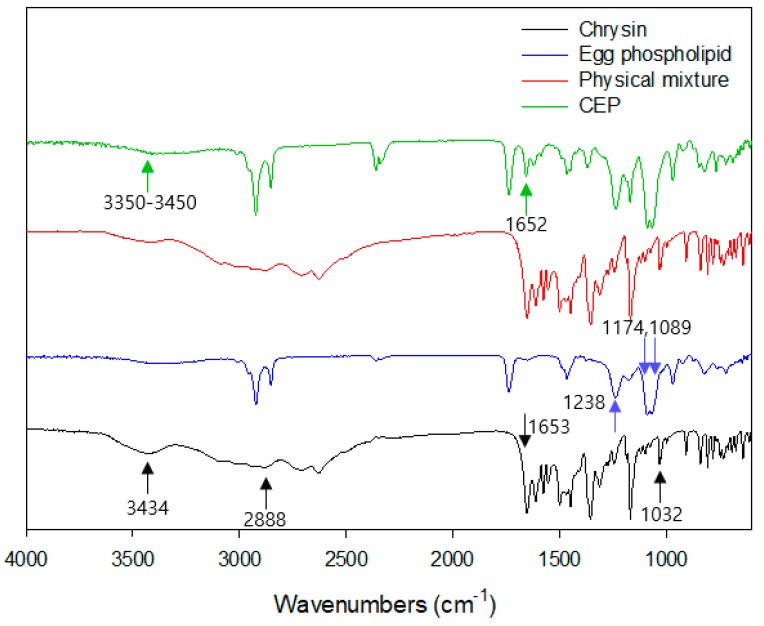
Fourier-transform infra-red spectra of chrysin, egg phospholipid, physical mixture, and CEP-1:3.

A FTIR spectroscopy study was performed to identify functional groups that involved in complex formation between chrysin and EPL. As shown in Figure 3, chrysin showed characteristic absorption peaks at 3434 cm^−1^, 2888 cm^−1^, 1653 cm^−1^, and 1032 cm^−1^ which are assigned to terminal –OH, C–H, C=O, and C–C (C–O and C–O) stretch, respectively. EPL displayed typical P=O and P–O–C peaks of the phosphate group at 1238 cm^−1^, 1174 cm^−1^, and 1089 cm^−1^. The peak indicating (CH_3_)_3_N stretching was found at 970 cm^−1^ as reported by Nzai and Proctor [34]. The absorption peak of the physical mixture was the sum of the characteristic chrysin and EPL peaks, while the phenolic -OH peaks (3350–3450 cm^−1^) of chrysin were decreased in CEP-1:3. In addition, the peak of the C=O group of the chrysin near 1652 cm^−1^ shifted to a higher wavelength. These changes reflect the involvement of phenolic OH and C=O groups in complex formation. Also, their hydrogen bonding counterparts, such as P=O and P–O–C peak signals, were weakened in the CEP-1:3 peak spectrum. Taken together, the overall FTIR peaks of CEP-1:3 was different from that of the physical mixture and more similar to that of EPL. This result suggests that that characteristic chrysin absorption peaks were shielded by EPL via complex formation.

Hou et al. [19] reported that the hydrogen bond was a major driving force for molecular complexation in a mitomycin-loaded phytosome, and other chemical bonds were not found in the complex. Non-polar flavonoids such as chrysin, are likely to be located in the hydrophobic region of phosphatidylcholine. Notably, the number of hydroxyl groups in the B-ring determines the strength of interaction between flavonoid and phospholipid [35]. Although hydrogen bond formation was a primary contributor to molecular complexation, hydrophobic interactions may have contributed to the affinity of chrysin for phospholipids. 

### 3.6. X-ray Diffraction (XRD) 

**Figure 4:**
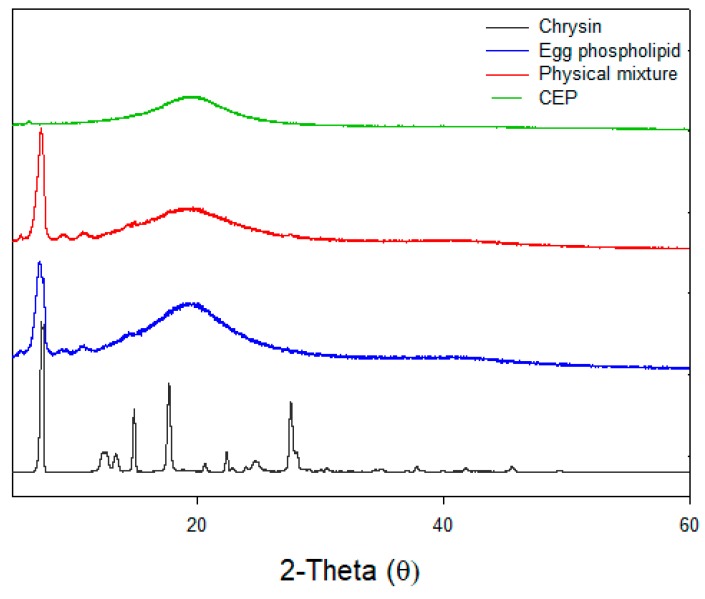
X-ray diffractograms of chrysin, egg phospholipid, physical mixture and CEP-1:3.

The XRD patterns of samples are shown in Figure 4. Chrysin yielded characteristic sharp crystalline peaks at 7.28 °C, 14.92 °C, 17.70 °C, and 27.60 °C in XRD, while a smooth broad peak diffractogram was found for the EPL. For the physical mixture, some characteristic crystalline diffraction pattern was still noticed; however, it was changed to a single peak at 2 θ = 19.18 °C. This confirms that chemical complexation between chrysin and EPL took place during the preparation of CEP as demonstrated by the result of the FTIR spectroscopic analysis. Moreover, the decreased intensity of the crystalline peak implied that a very limited amount of free chrysin remained in the CP. The loss of crystallinity and conversion to an amorphous state is a common structural feature of phytosomes [36].

### 3.7. Scanning Electron Microscopy (SEM) 

**Figure 5:**
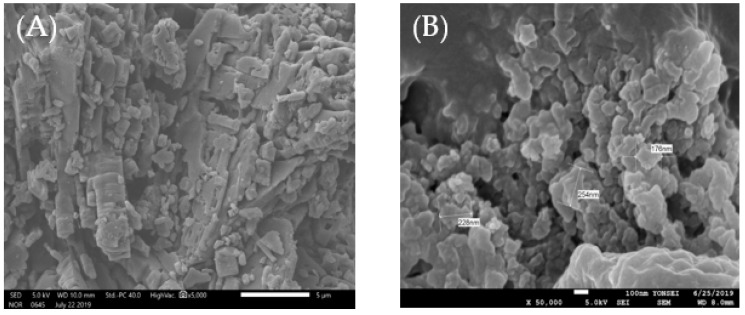
Scanning electron micrographs of (**A**) chrysin and (**B**) CEP-1:3.

The morphological characteristics of the solid state of CEP were visualized using SEM. As shown in Figure 5, chrysin was visualized as rod-shaped crystals; however, amorphous (170–250 nm) spherical particles were found in CEP-1:3. This finding confirmed the result of the XRD analysis, indicating that the crystalline state of chrysin was converted to an amorphous state through phospholipid complexation.

### 3.8. In Vitro Release of CEP

**Figure 6:**
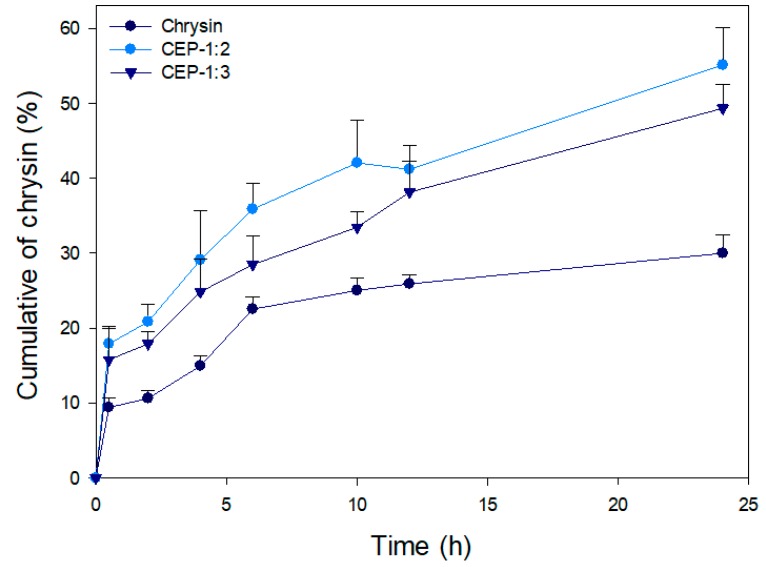
In vitro release profile of chrysin-loaded phytosomes.

The cumulated release profiles of samples were analyzed. When chrysin was dispersed in PBS, its dissolution/release from the dialysis bag within 24 h was very limited (<8%, data not shown). Samples were initially dissolved in methanol and the time-dependent release patterns were compared to obtain better insight into the release characteristics of chrysin and CEP. CEP-1:2 and CEP-1:3 resulted in faster and continuous release patterns versus chrysin (Figure 6). The release of chrysin reached a plateau in 6 h, and further release was not observed. This is probably attributed to the gradual decrease in the solubility of chrysin in PBS as dialysis proceeded. The cumulated amount of released chrysin from CEP-1:2 and CEP-1:3 after 24 h was 52% and 43%, respectively. Of note, they showed a continuous release trend even after 24 h. The improved aqueous solubility and transition to an amorphous structure through phospholipid complexation (Figure 1 and Figure 4) probably contributed to the positive changes in release behavior.

Samples dissolved in methanol (40%) were placed in a dialysis bag and the amount of chrysin released into the PBS buffer (pH 6.7) was quantified. Hou et al. [19] reported that the speed of release varies depending on the strength of phytochemical–phospholipid complex in phytosomes. The sustained release pattern of CEP-1:3 compared with that of CEP-1:2 may be partly explained by the formation of a relatively stable complex. Mancini et al. [37] compared the release characteristics of an *Annona muricata* extract-loaded phytosome and liposomes. They concluded that both nano-formulations retained their antioxidant activity. However, the phytosome resulted in faster extract release and was less toxic versus the liposomes. Based on the results, chrysin–phospholipid complexation imparts greater aqueous solubility by providing the amphiphilic nature of phospholipids, in turn facilitating prolonged release as compared to free chrysin.

### 3.9. Effect of CP on Glucose Uptake in C2C12 Skeletal Muscle Cells

**Figure 7:**
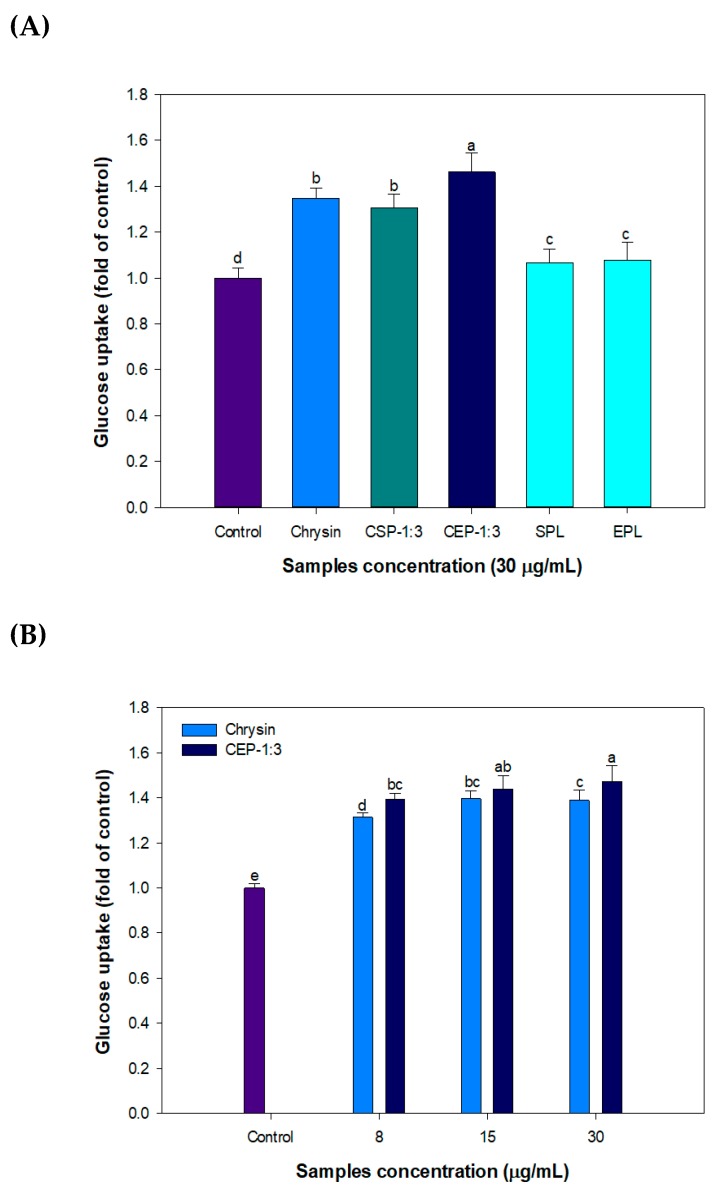

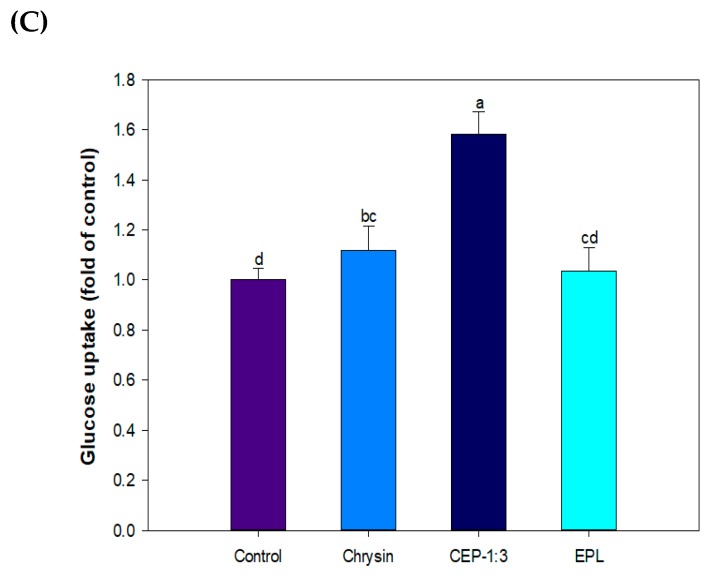
Effect of (**A**) phospholipid matrix, (**B**) dose-dependent behavior, and (**C**) sample filtration on glucose uptake in C2C12 skeletal muscle cells. (A) Chrysin, CSP-1:3 and CEP-1:3 were fully solubilized using DMSO (1%, *v/v*), (B) The dose-dependent effects of samples were compared. (C) Chrysin and CEP-1:3 obtained after filtration of samples (equivalent to 1 mg/mL chrysin) through a 0.45 μm syringe filter. All samples were treated for 24 h and glucose uptake was determined. Bars with different letters indicate significant differences at (*p* < 0.05).

The effects of CSP-1:3 and CEP-1:3 on glucose uptake were examined using C2C12 skeletal muscle cells. Chrysin treatment (30 μg/mL, 1% DMSO) significantly increased glucose uptake compared with control and CEP-1:3 exerted the highest glucose uptake promoting effect. Treatment with empty phytosomes showed higher glucose uptake than control but their effects were limited compared with that of chrysin (Figure 7A). CEP-1:3 improved glucose uptake in a dose-dependent manner (Figure 7B). The effect of chrysin and CEP-1:3 on glucose uptake was examined after filtration of excess amount of sample (1 mg/mL) using a 0.45 μm syringe filter. As shown in Figure 7C, the difference in glucose uptake between chrysin and CEP-1:3 was increased after filtration of samples.

The absorption of chrysin into C2C12 skeletal muscle cells may be critically influenced by two major factors, namely aqueous solubility and the capability of chrysin to diffuse across cell membranes. The lipophilic nature and increased aqueous solubility of CEP are probably responsible for the improved glucose uptake. Interestingly, CSP-1:3 was not as effective as CEP-1:3 on glucose uptake and this was possibly due to differences in aqueous solubility (Figure 1) and phytosome structure (Figure 2).

Phospholipids may provide multiple functionalities and act as carrier and active biomolecules. SPC selectively inhibited Toll-like receptor 4-mediated inflammatory signaling such as the expression of monocyte chemoattractant protein-1, in vascular cells induced by lipopolysaccharide and palmitate [38]. However, the contribution of EPL itself on glucose uptake in C2C12 muscle cells was not clearly demonstrated in this study.

### 3.10. Effect of CEP-1:3 on the Expression of Genes Related to Glucose Uptake in C2C12 Skeletal Muscle Cells

**Figure 8:**
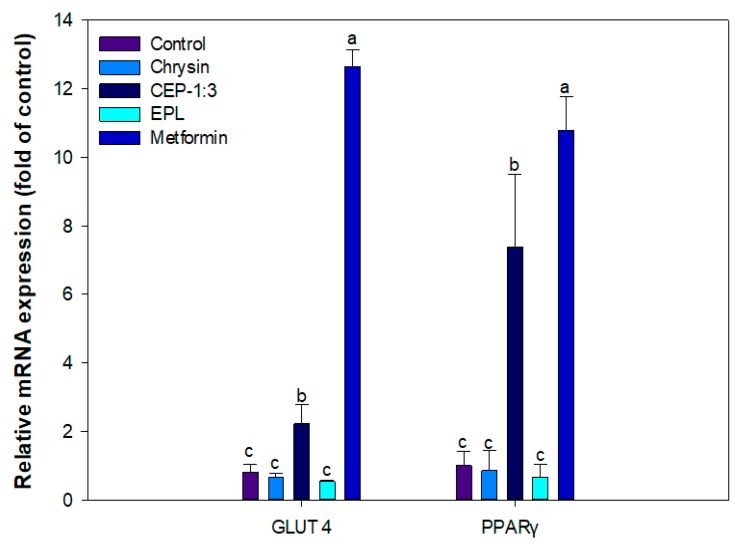
Effects of chrysin and CEP-1:3 on the expressions of PPARγ and GLUT4 genes in C2C12 skeletal muscle cells**.** Chrysin and CEP-1:3 obtained after filtration of samples (equivalent to 1 mg/mL chrysin) through a 0.45 μm syringe filter. The mRNA expression levels of PPARγ and GLUT 4 genes were analyzed using qPCR and normalized to that of β-actin. Bars with different letters indicate significant differences (*p* < 0.05).

The effects of samples on the expression of peroxisome proliferator-activated receptor γ (PPAR γ) and glucose transporter type 4 (GLUT4) genes were analyzed to find to identify major contributor to CEP-1:3-mediated improved glucose uptake in C2C12 muscle cells. qRT-PCR analysis indicated that CEP-1:3 upregulated the mRNA expression of PPARγ and GLUT4 while chrysin obtained after filtration did not show significant difference in PPARγ expression versus control (Figure 8). The treatment with empty phytosome did not show significant effect on gene expression of PPARγ and GLUT4.

PPARγ is a ligand-activated transcriptional factor which modulates glucose homeostasis. The activation of PPARγ is an established therapeutic target for diabetes. Feng et al. [39] reported that chrysin treatment selectively stimulated the expression of PPARγ and attenuated inflammation in macrophages and mice fed a high fat diet. Dhaunsi et al. [40] reported that decreased activation of PPARγ was associated with increased renal arterial injury in diabetic animals. Verma et al. [41] reported that modulation of PPARγ expression in C2C12 muscle played a critical role in the attenuation of type 2 diabetes, and overexpression PPARγ expression in muscle can be an effective pharmacological target against insulin resistance. Glucose uptake in muscle is mediated by insulin dependent IRS/PI3K/AKT and/or insulin independent AMPK signal transduction. In both cases, translocation of GLUT4, a major glucose transporter, translocates to the plasma [42]. The expression of GLUT4 and its plasma translocation was significantly suppressed in the diabetic state [43]. Satyanarayana et al. [44] reported that chrysin improved blood glucose control via activation of insulin signaling in high fat induced diabetic rats.

Several phytochemicals, such as chlorogenic acid and berberine, upregulated the expression of PPARγ and GLUT4, and increased glucose uptake in myotubes. It has been suggested that these phytochemicals display a synergistic effect with commercial hypoglycemic drugs such as metformin [45].

Based on these speculations, the stimulated gene expressions of PPAR and GLUT4 by CEP-1:3 were responsible for the improved glucose uptake noted in muscle cells.

## 4. Conclusions

Complexation of chrysin with phospholipids improved solubility and promoted glucose uptake in C2C12 muscle cells. However, the extent of improvement varied depending on the type of phospholipid matrix used for the preparation of phytosome. EPL consisting of various phospholipid species produced elastic and stable CPs compared to those produced with SPC. The nanoformulated CEP-1:3 displayed a greater glucose uptake promoting effect than free chrysin and CSP-1:3 in muscle cells by stimulating the expression of PPARγ and GLUT4 genes. The difference in glucose uptake-promoting effect between free chrysin and CEP-1:3 was increased when filtered samples were used. The results of the present study suggest that the phospholipid matrix used for the preparation of phytosomes critically influences their performance.

## Figures and Tables

**Table 1:** Average particle size, polydispersity index (PDI), zeta potential, and encapsulation efficiency of chrysin-loaded phytosomes.

	Average Particle Size (nm)	PDI	Zeta Potential (mV)	Encapsulation Efficiency (%)
CSP-1:2	129 ± 2.0	0.45 ± 0.02	−28 ± 0.2	98.8 ± 0.1
CSP-1:3	134 ± 0.1	0.49 ± 0.01	−33 ± 4.1	98.6 ± 0.1
CEP-1:2	89 ± 0.5	0.25 ± 0.01	−21 ± 0.3	98.8 ± 0.1
CEP-1:3	117 ± 0.7	0.29 ± 0.02	−31 ± 0.8	98.7 ± 0.1
Storage for 21 days at 4 °C				
CEP-1:3	118 ± 1	0.30 ± 0.02	−32 ± 0.3	

CSP-1:2 and CSP-1:3, chrysin-loaded phytosome prepared with soya phosphatidylcholine at the molar ratio of 1:2 and 1:3, respectively. CEP-1:2 and CEP-1:3, chrysin-loaded phytosome prepared with egg phospholipid at the molar ratio of 1:2 and 1:3, respectively.

**Table 2:** Elastic moduli of CSP-1:3 and CEP-1:3.

	CSP-1:3	CEP-1:3
Elastic modulus (kPa)	12.4 ± 0.52	33.2 ± 0.90

The elastic modulus of samples was measured using AFM equipped with a colloidal probe (colloid size: 10 μm) with a gold coating on the reflex side and a spring constant of 0.14 N m^−1^. CSP-1:3, chrysin-loaded phytosome prepared with soya phosphatidylcholine at the molar ratio of 1:3; CEP-1:3, chrysin-loaded phytosome prepared with egg phospholipid at the molar ratio of 1:3.
